# Viewer Engagement in Response to Mixed and Uniform Emotional Content in Marketing Videos—An Electroencephalographic Study

**DOI:** 10.3390/s24020517

**Published:** 2024-01-14

**Authors:** Izabela Rejer, Jarosław Jankowski, Justyna Dreger, Krzysztof Lorenz

**Affiliations:** 1 Department of Computer Science and Information Technology, West Pomeranian University of Technology in Szczecin, 70-310 Szczecin, Poland; jaroslaw.jankowski@zut.edu.pl (J.J.);; 2 Krzysztof Lorenz Institute of Economics and Finance, University of Szczecin, 70-453 Szczecin, Poland; krzysztof.lorenz@usz.edu.pl

**Keywords:** EEG, engagement index, emotional content, marketing videos

## Abstract

This study presents the results of an experiment designed to investigate whether marketing videos containing mixed emotional content can sustain consumers interest longer compared to videos conveying a consistent emotional message. During the experiment, thirteen participants, wearing EEG (electroencephalographic) caps, were exposed to eight marketing videos with diverse emotional tones. Participant engagement was measured with an engagement index, a metric derived from the power of brain activity recorded over the frontal and parietal cortex and computed within three distinct frequency bands: theta (4–8 Hz), alpha (8–13 Hz), and beta (13–30 Hz). The outcomes indicated a statistically significant influence of emotional content type (mixed vs. consistent) on the duration of user engagement. Videos containing a mixed emotional message were notably more effective in sustaining user engagement, whereas the engagement level for videos with a consistent emotional message declined over time. The principal inference drawn from the study is that advertising materials conveying a consistent emotional message should be notably briefer than those featuring a mixed emotional message to achieve an equivalent level of message effectiveness, measured through engagement duration.

## 1. Introduction

Online consumption of video content is currently prevalent and is continuing to grow at a significant rate. This growing trend creates a natural opportunity for advertisers to integrate video ads with the primary video content in the form of in-stream video ads [1]. However, this integration presents a challenge for advertisers to create ads that can effectively capture and maintain the attention of consumers. As a result, there is a growing interest in identifying factors that impact video ad performance [2], particularly those that can help capture and maintain user attention [3].

The problem is that advertising content that fails to catch consumers’ attention at first glance is most likely to be immediately skipped. Therefore, ad producers face the challenge of creating video ads in a way that decreases the likelihood of consumers skipping them. One factor that significantly contributes to this idea is consumer engagement. As consistently reported by different authors, consumers who are more engaged in video content are less likely to skip it [4,5]. Other studies in the field have also underlined the relationship between ad avoidance and cognitive categories closely related to engagement. For example, in [6], it was found that ad content avoidance may be related to high and low arousal levels, evoked by the content. A similar observation was reported in [7], where it was found that boring and unengaging content increases the intention of skipping ads.

The engagement in marketing content appears to be the primary factor influencing consumers’ tendency to avoid ads. Another crucial factor that impacts ad avoidance is the in-video length [7]. The relationship between the rate of ad avoidance and in-video ad length has been discussed in several studies, which have identified a correlation between increased skipping behavior and longer marketing content [7,8]. Other research suggested that longer ads result in higher levels of disruption for goal-oriented searches [9,10].

Recent studies have shown that the acceptance level for extended videos has declined, and nowadays, the majority of consumers accept only extremely short video ads, with lengths of fifteen or even six seconds [7]. Therefore, the current trend is to produce short video marketing content to better target consumers’ attention spans. On the one hand, this trend is positive for ad providers since they can increase the rate of presenting content to consumers without increasing the total cost of a campaign. On the other hand, some studies have shown that longer ads may be more effective and enhance brand recognition [8]. Advertisements shortened to fifteen seconds can deliver similar results to thirty-second-long spots in terms of awareness and brand recall. However, thirty-second ads result in stronger persuasive and emotional effects [11].

Hence, one advantage of longer video ads is the ability to convey emotions. This is important for ad providers because emotions are known as an essential factor for increasing consumer engagement, which directly translates to an increased effectiveness of video ads [12]. The impact of emotions incorporated into video content on consumer behavior has been analyzed from various perspectives, including behavioral engagement [12], content sharing [13], and consumer attitudes [14]. For example, recent studies have demonstrated a positive correlation between surprise, joyfulness, and the level of consumer attention and retaining target audiences [15].

In addition to emotional tone, several other content features have been identified as significant factors in maintaining user engagement, such as language, linguistic style, subjectivity, and video category [16]. In this paper, we present a study that analyzes another factor that may influence consumer engagement: the emotional style of the content. We assume that although emotions are crucial for sustaining consumer engagement in the presented content, emotionally uniform content has a lower potential to maintain consumer engagement for an extended period compared to content that elicits various emotions over time. Our assumption is based on the fact that emotionally uniform content may lead to attentional habituation, which is typical for continuous stimuli [17]. According to [18], the amount of attention paid to emotional content decreases as the time of presenting the content increases. Since the goal of affective habituation is to promote flexibility and prevent panic-like states, as suggested by [19], responses to positive content decrease faster than those to fearful content, which is the result of a stronger response to signals that inform users about dangerous situations [20]. From a neuroscience point of view, the habituation to emotional content is associated with decreased activity in the amygdala and prefrontal cortex, two brain regions that are responsible for processing and regulating emotions [21].

As was mentioned above, attentional habituation occurs as a consequence of exposure to emotionally uniform content. In this paper, we postulate that manipulating the emotional content of a marketing video by introducing changes in the emotions elicited could impede the development of habituation and thus prolong consumer attention compared to videos with uniform emotion. In other words, we hypothesize that the use of mixed emotions could increase consumer engagement with video content by mitigating the habituation effect. If our hypothesis holds, the use of mixed emotions in video advertisements may prove to be a crucial factor in decisions to extend the length of video ads, which is associated with increased empathic response to ad content and, consequently, with an increased effectiveness of marketing campaigns [22]. Additionally, according to [10], while uniform emotional content may increase ad-skipping rates, complex emotional responses such as humor or nostalgia can enhance performance and sustain users’ interest.

Various studies have analyzed the impact of mixed emotions on ad performance. Concerning ad recall, mixed emotions were found to be more difficult to accurately recall than uniform emotions, and it was also observed that they might induce a quicker decay effect [23]. However, in terms of impact on user behavior, results are dependent on the area of application and content type. For instance, in the case of viral content, intentions to follow videos with mixed emotions were shown to be lower than those for videos with uniform emotions and positive tones. Users were simply less interested in emotionally unstable content [24]. Conversely, the authors of a recent study related to word-of-mouth marketing (WOM) reported that a mixed emotional appeal is more effective than pure happiness when combined with third-person narration [25]. Additionally, it was observed that mixed emotional content has the potential to deliver a more positive user experience than pure positive content. For example, humorous elements mixed with fear increase ads’ effectiveness through greater persuasive potential [26]. Earlier studies also showed that mixed emotions could be used to increase ad effectiveness through persuasive appeal [27]. For instance, negative scenes followed by positive content lead to conflicting psychological states. In the sector of pro-environmental luxury companies, mixing happiness and sadness increases intention to purchase compared to happiness only [28]. Lastly, mixed emotions incorporated into video content have a higher impact on post-message consumers’ behavior than uniform emotions [29].

The studies mentioned earlier have examined the impact of mixed emotions on video ad effectiveness using measures such as persuasion, recall, and intention to purchase. However, the objective of the present study was to investigate this relationship in terms of consumer engagement using the most direct measure possible: the direct analysis of brain activity recorded during the watching process. The study analyzed two types of marketing videos: those with a clear and uniform emotional message and those with more complex emotional content. It was assumed that due to habituation, uniform videos would maintain viewer engagement for a significantly shorter period than videos with mixed emotional content. Thus, the primary hypothesis of the study was that marketing videos with a mixed emotional content maintain subjects’ engagement for a longer period than videos with consistent emotional messages.

Methods used for determining the level of subjects’ engagement can be broadly classified into three categories: (i) self-reporting questionnaires, (ii) behavioral measures based on task performance, and (iii) measures based on the physiology of users [30]. The recognition of engagement levels based on self-reporting and behavior-based information tends to be delayed, sporadic, and intrusive [31]. Additionally, performance-based measures can be misleading, since multiple degrees of engagement might correspond to the same level of performance [30]. In contrast, physiological measures are available at any time and have significantly shorter delays measured on a scale of seconds. They are also independent of the subjects’ opinions, claims, and imaginations, which allows for the objective evaluation of engagement levels. Although different physiological measurements, such as electrocardiography (ECG), galvanic skin responses (GSRs), electromyography (EMG), or eye movements (ET) [32], have been studied, measurements reflecting the subject’s brain activity, such as electroencephalography (EEG) and near-infrared spectroscopy (NIRS) [30,31,33], provide the most direct information about a subject’s engagement level.

Engagement indexes based on features derived from electrical brain activity have been used to assess subjects’ engagement levels in many different types of tasks, both cognitive and physical. For example, in [34], different engagement metrics based on EEG activity were studied to assess the engagement level for a set of cognitive tasks (grid location, mental arithmetic, forward and backward digit span, and trail-making task). A similar study, though only focused on three tasks (mental arithmetic and forward and backward digit span), was reported in [30]. On the other hand, in [35], engagement level was used as a predictor that enabled the recognition of cognitive task demands. The five tasks applied in that study were baseline with eyes opened, multiplication, letter composition, geometric figure rotation, and visual counting.

In addition to psychological experiments, EEG engagement metrics have been used in many real-life studies. For example, in [36], EEG engagement estimates were used to predict the success or failure of solving math problems. Similarly, in [37], the EEG engagement ratio, along with other indexes, was used to monitor the mental state of pilots in a flight simulator and an actual light aircraft. In [38], players’ engagement was evaluated while playing the Super Meat Boy platform game, and in [39], engagement level was monitored to improve stroke rehabilitation effectiveness.

The EEG correlates of engagement level are well documented in the neurophysiological literature [40]. It is generally believed that an increase in beta activity reflects a higher degree of alertness and greater engagement in the task, whereas an increase in alpha and/or theta activity reflects less alertness and lower task engagement [41]. Based on this assumption, various engagement indexes have been established and tested in the underlying studies [41,42,43,44]. Among these indexes, the most commonly used is the engagement index (EI), which directly correlates engagement with beta activity and inversely correlates engagement with alpha and theta activity [39,41,42,45,46]. This index was also applied in our survey. Following the methodology outlined in [44], we calculated the index values using EEG signals recorded over the parietal and frontal cortex (specifically, at F7, F3, Fz, F4, F8, P3, Pz, and P4).

To verify our research hypothesis, we recruited 13 participants to watch a series of eight videos with varying emotional content. While five videos featured emotionally stable content (including two happy, two sad, and one neutral video), the remaining three videos were designed to elicit diverse emotional responses across different scenes. The main outcome of the experiment indicated a notable decrease in participants’ engagement over time when viewing emotionally stable videos, regardless of emotional direction. Conversely, despite their longer duration in comparison to uniform videos, those with mixed emotional content evoked a progressive increase in participants’ engagement levels over time.

This paper is organized as follows. Section 2 outlines the experimental setup and details the method used for calculating the engagement index. In Section 3, we present the results of the experiment. In Section 4, we provide a detailed discussion of the results in the context of previous research in the field. Finally, Section 5 concludes the paper.

## 2. Materials and Methods

### 2.1. Experiment Setup

To investigate the impact of marketing content length and emotional load on individuals’ responses, we conducted an experiment involving 13 healthy, right-handed participants with no history of mental disorders. The recruitment process was carried out at the West Pomeranian University of Technology in Szczecin. Targeting the university’s academic community, the recruitment pool comprised both university students and academics. Prospective participants were initially contacted through official university channels and then a screening process was implemented to assess eligibility. The inclusion criteria included individuals with no history of neurological or psychiatric disorders, absence of medication affecting central nervous system activity, and proficiency in the Polish language. Exclusion criteria encompassed any contraindications for EEG recording, such as the presence of metallic implants or severe skin conditions. The recruitment process was carried out between January and March 2021. The detailed demography of participants recruited to the study is presented in Table 1.

The task assigned to the participants was to view eight videos, each featuring different marketing content, displayed on a computer screen. Immediately after watching each video, participants were required to report their attitude toward the presented content by selecting one or more items from a list of eight available options: happiness, sadness, surprise, excitation, anger, fear, disgust, and calmness. Additionally, four questions asking for details of the last viewed video were prepared for each video. Participants were presented with the questions after they had finished describing their emotional state.

**Figure 1:**
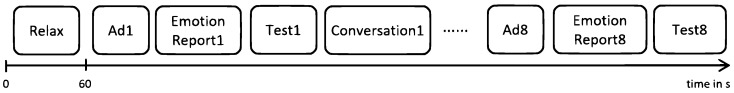
Detailed scheme of the experiment.

The videos were presented in a random order, one after the other. To help participants overcome the emotions evoked by the presented content, a “neutral” conversation was held between the participant and the experimenter after each video. During this conversation, the subject’s engagement index was monitored and the next video was displayed only when the index remained for about a minute on a sufficiently small level. Prior to the first video, participants were asked to relax for one minute while viewing a neutral gray background displayed on the screen. A detailed overview of the experiment can be found in Figure 1. The duration of the experiment for each participant was approximately 50–70 min, in addition to the time required to apply and remove the EEG cap. To encourage participants to pay close attention to the presented content, the experiment was held in the form of a competition with a prize awarded to the three participants with the highest scores.

### 2.2. Videos

To facilitate understanding of the results reported in the Results section, a brief description of the eight videos viewed by the participants during the experiment is provided in Table 2. Additionally, Table 3 summarizes the key characteristics of each video. As can be noticed in Table 2, three of the videos used in the experiment were commercial ads, while the remaining five were social campaigns. For the four social campaigns, the final parts containing only text written on the screen were excluded. The numbers in parentheses in the third column of Table 3 indicate the duration of the video portions omitted during the analysis.

### 2.3. EEG Signal Processing

EEG data were recorded from 19 monopolar channels with passive gold gel electrodes at a sampling frequency of 500 Hz. Electrode placement adhered to the International 10–20 system guidelines established by Jasper in 1958 [55]. The ground electrode was affixed at Fpz, while the reference electrode occupied the left mastoid position. The impedance was maintained below five kΩ across all channels. The signal was acquired with a MITSAR 202 amplifier, recorded with Mitsar EEG Studio Acquisition 1.9 software, and processed and analyzed in a Matlab environment.

**Figure 2:**
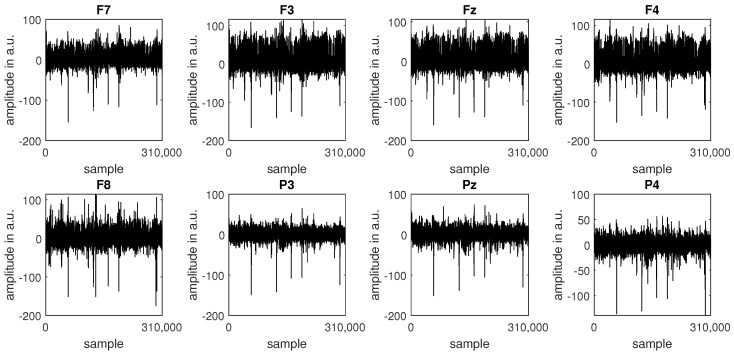
Raw EEG data for subject S1.

**Figure 3:**
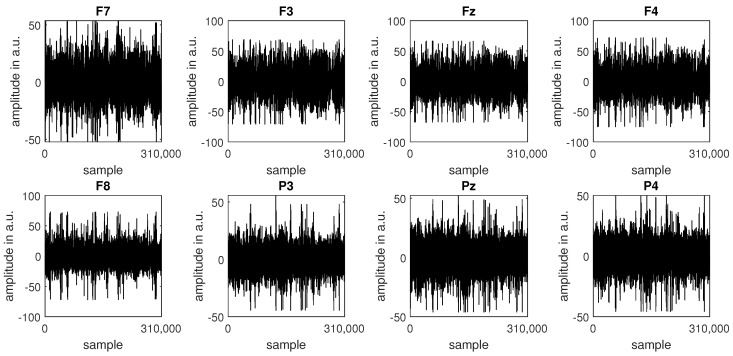
The EEG data for subject S1 after the three-step filtering procedure.

At the beginning of the signal pre-processing stage, nine epochs corresponding to a “relax” event and eight videos were extracted from the complete recording. Next, the epochs were concatenated, and a set of IIR filters was applied to clean the signal and prepare it for analysis. First, a pair of Butterworth temporal filters of the 4th order was used to attenuate the signal outside the 0.5–30 Hz range (a high-pass filter with a cutoff frequency of 0.5 Hz and a low-pass filter with a cutoff frequency of 30 Hz). Second, a spatial filter was applied to address EOG (electrooculographic) artefacts. Spatial filtering was conducted with a MATLAB implementation of the FastICA [56] algorithm, one of the algorithms used to solve an ICA (independent component analysis) model. Finally, a statistical median filter with a kernel length of 100 samples was employed to eliminate episodic spiking artefacts (outliers). The outliers’ thresholds were set to the 0.1 and 99.9 percentiles. Figure 2 and Figure 3 present an example of raw (Figure 2) and cleaned (Figure 3) signals recorded from subject S1.

### 2.4. Engagement Index

The cleaned signal was again split into the primary epochs. Then, since we aimed to investigate the engagement dynamics, we further subdivided these epochs into windows with a five-second duration, with four-second overlaps. For each window, we extracted 24 band power features comprising the power in theta (4–8 Hz), alpha (8–13 Hz), and beta (13–30 Hz) bands calculated for eight channels. Five of those channels were positioned over the frontal cortex (F7, F3, Fz, F4, and F8), while the remaining three were situated over the parietal cortex (P3, Pz, and P4). After obtaining the power for the given EEG bands, the relative power was computed for each band and each channel:

(1) $$\begin{array}{c} ThetaR=\frac{power_{Theta}}{power_{Theta}+power_{Alpha}+power_{Beta}} \end{array}$$

(2) $$\begin{array}{c} AlphaR=\frac{power_{Alpha}}{power_{Theta}+power_{Alpha}+power_{Beta}} \end{array}$$

(3) $$\begin{array}{c} BetaR=\frac{power_{Beta}}{power_{Theta}+power_{Alpha}+power_{Beta}} \end{array}$$

**Figure 4:**
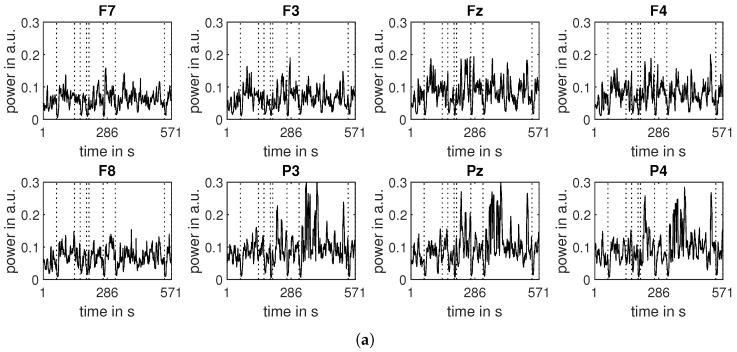

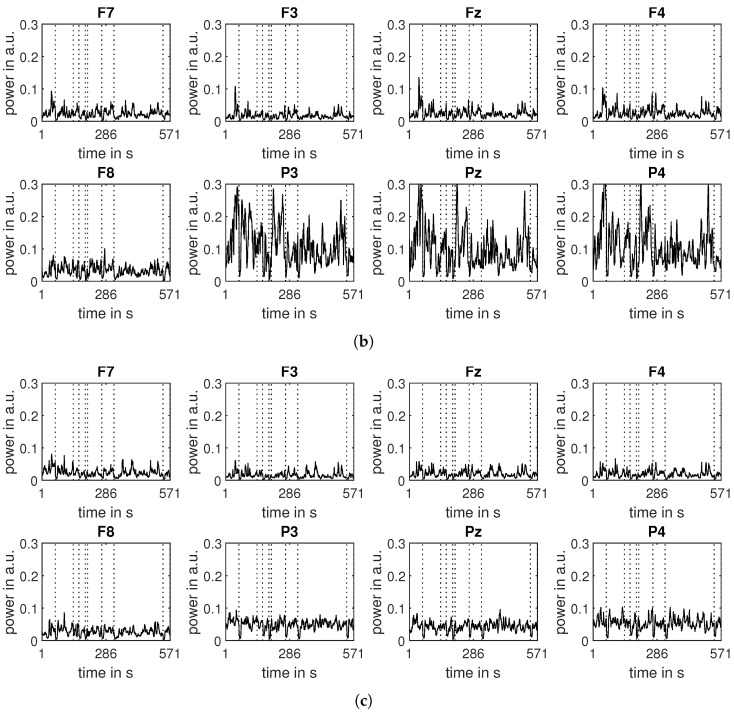
Relative power in three frequency bands calculated for all time windows for subject 1; dotted vertical lines separate consecutive epochs; (**a**) Frequency band: theta (4–8 Hz); (**b**) Frequency band: alpha (8–13 Hz); (**c**) Frequency band: beta (13–30 Hz).

Figure 4 presents power band features calculated for all time windows for Subject 1.

The relative band power features were used to calculate the engagement index:

(4) $$\begin{array}{c} E=\frac{sum(BetaR(F7,F3,\cdots ,P4))}{sum(AlphaR(F7,F3,\cdots ,P4))+sum(ThetaR(F7,F3,\cdots ,P4))} \end{array}$$

**Figure 5:**
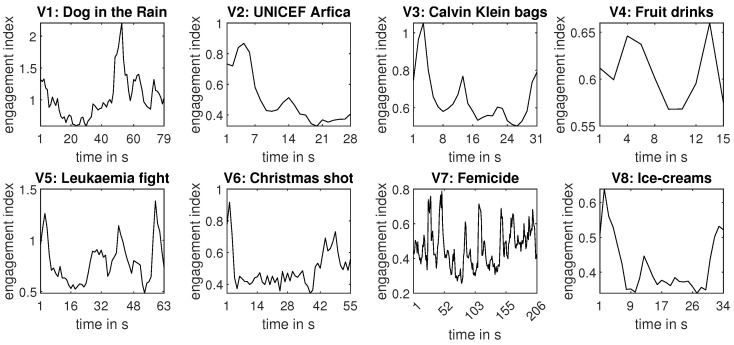
Engagement indexes obtained for subject S1 for all video events.

**Figure 6:**
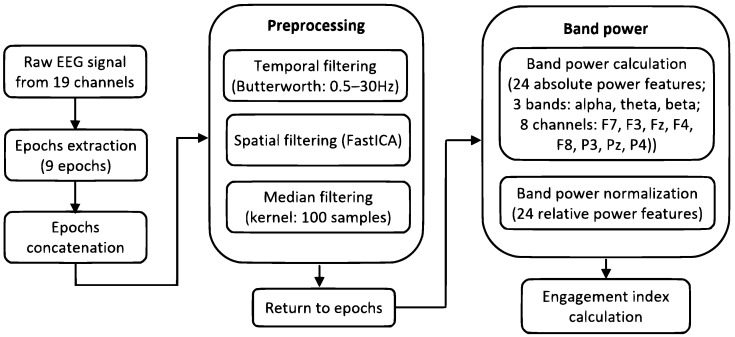
Signal processing chain.

The engagement index was calculated for the consecutive windows of each event. Figure 5 presents a set of indexes obtained for all video events for a single subject (S1), and Figure 6 summarizes the whole signal processing chain from raw EEG signals to engagement indexes.

## 3. Results

Prior to analyzing the EEG data collected during the experiment, we first examined the emotional responses provided by the subjects to find out whether they aligned with our assumptions. Table 4 presents the total number of subjects reporting each type of emotion for each video. It is important to note that since subjects could choose more than one emotional response for a given video, the total number of emotions reported by all subjects for each video may have exceeded the number of subjects.

The final column of Table 4 reveals that for videos classified as ‘mixed’, much more emotions were ticked by subjects compared to videos classified as ‘consistent’. This trend is particularly noticeable for videos V6 and V7, for which the total number of reported emotions was twice as high than all ‘consistent’ videos (apart from V5). Furthermore, while the dominant emotions for ‘consistent’ videos were indeed consistent (either sad or happy/calm), this was not the case for ’mixed’ videos. For instance, in videos V6 and V7, sadness (a low-arousal emotion associated with withdrawal motivation) was mixed with anger (a high-arousal emotion associated with approach motivation). In the case of V6, sadness and anger were accompanied by surprise, whereas in V7, they were accompanied by fear and disgust. An even greater emotional mix was observed for V1, where subjects reported both happiness and sadness.

Upon comparing the emotions reported by the subjects to the emotions assigned to each video based on the soundtrack analysis, we found that for most ’consistent’ videos, the emotions reported by the subjects were consistent with our assumptions. However, for ’mixed’ videos, our predictions were not as accurate. For example, in V1, we correctly predicted sadness and surprise, but subjects reported happiness instead of anger. In V6, we forecasted anger, happiness, and calmness, but subjects reported anger, sadness, and happiness. Finally, in V7, we accurately predicted fear, sadness, and anger, but subjects also reported disgust.

We commenced the analysis of the EEG signals by examining whether there was a significant difference in the mean level of engagement among video events. As the distribution of data for some events was skewed towards the right, we transformed the data using a logarithmic function. Subsequently, Lilliefors normality tests were performed with a *p*-value of 0.001, indicating that the hypothesis of normality for each group (video) could not be rejected. ANOVA statistics were then applied to test the significance of differences among conditions. Given that the data streams were unequal for different videos, a one-way unbalanced ANOVA design was used with a factor VIDEO (eight levels). The significance level was set to 0.01 for this and all subsequent tests reported in this paper.

**Figure 7:**
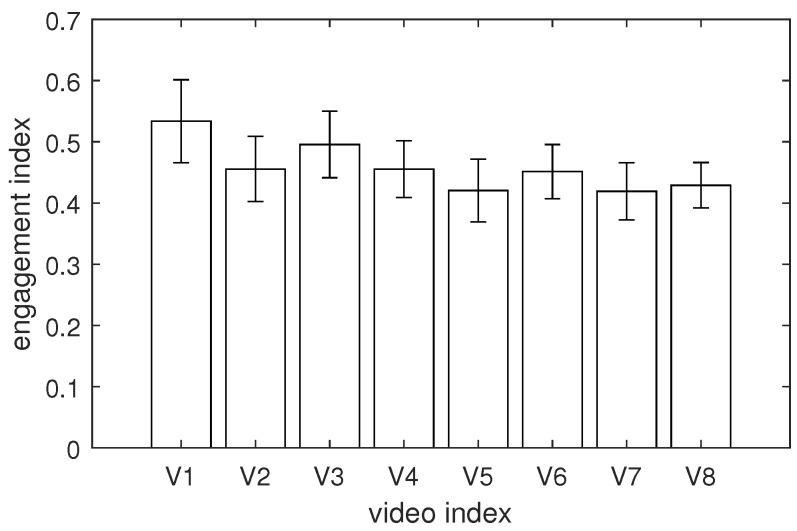
The average engagement index calculated for each video (error bars denote standard deviations).

The test returned *F* = 33.46 with a *p*-value = 0, indicating that not all group means were equal. To identify which pairs of means were significantly different, we conducted a set of *t*-tests with Bonferroni correction. Table 5 presents the outcomes of the post hoc tests performed for each pair of video events. The pairs of video events with a significant *p*-value (*p*-value < 0.01) are marked with an asterisk in the table. Additionally, Figure 7 illustrates the comparison of means calculated for each video. To ensure a reasonable interpretation of group means, the means shown in Figure 7 were calculated using the original, instead of log-transformed, data.

The pairwise comparisons showed that subject engagement was not consistent across all videos. The highest engagement level was recorded for videos V1 (mean = 0.534) and V3 (mean = 0.495). However, while the engagement level for V1 was significantly higher than for most other videos (V2, V5, V6, V7, and V8), for V3, the data dispersion was so large that a significant difference was observed only for two videos (V5 and V7). Additionally, significant differences were noted for video V6 (mean = 0.451)—the engagement level was significantly higher than two other videos, V5 and V7. Although the engagement level observed for video V4 was slightly higher (mean = 0.455) than that for video V6, no significant differences were found for video V4. The smallest engagement levels were observed for videos V5 (mean = 0.420), V7 (mean = 0.420), and V8 (mean = 0.429).

The results presented in Figure 7 did not allow for any conclusions about the differences between the videos with the mixed and consistent emotional loads since the two videos with the highest (V1 and V3) and lowest engagement levels (V5 and V7) belonged to opposite groups (V1 and V7—mixed; V3 and V5—consistent). Therefore, to provide direct insight into the differences between the videos with mixed and consistent emotional contents, we performed an additional one-way ANOVA test with a factor CONTENT of two levels: mixed and consistent. The classification of videos into one of the two groups was performed according to the labels presented in Table 3. Hence, group 1 (mixed content) contained three videos (V1, V6, and V7) and group 2 (consistent content) contained the remaining five videos (V2, V3, V4, V5, and V8).

**Figure 8:**
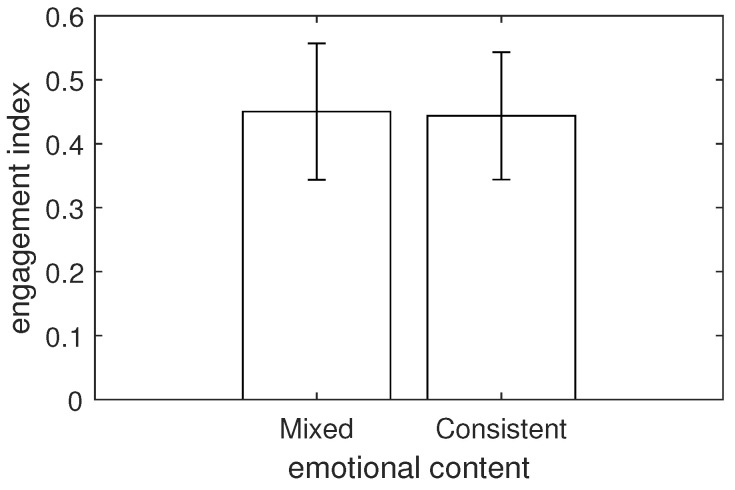
The average engagement index calculated over videos with mixed and consistent emotional contents (error bars denote standard deviations).

The test returned *F* = 1.137 with a *p*-value = 0.286, indicating no statistically significant difference in engagement level between videos with mixed and consistent emotional contents. A comparison of means is presented in Figure 8.

The global analysis presented above compared engagement levels across videos or content conditions during the entire analyzed period. Although it provided some insight into the subjects’ engagement in the presented content, its utility is limited. For an ad provider, merely capturing a consumer’s attention briefly at the beginning of a video is insufficient; it is crucial to sustain their interest until the end. A high engagement level at the end of a video increases the likelihood that the message will be retained in the consumer’s mind for longer. An even more favorable scenario for an ad provider is when consumer engagement not only remains high at the end of a video but continues to grow throughout the ad presentation. Therefore, to obtain more meaningful results, engagement levels need to be analyzed over time.

**Figure 9:**
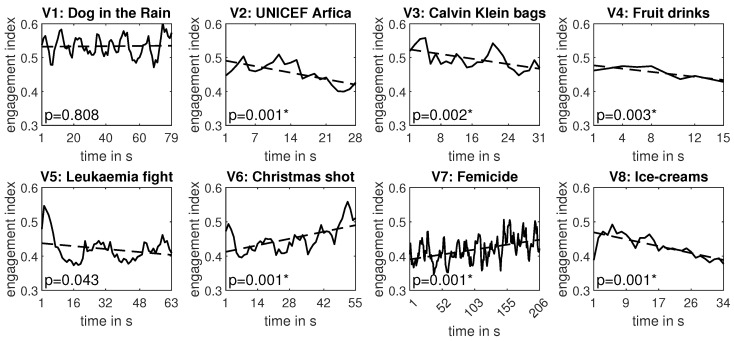
The engagement indexes averaged over subjects (solid curves) and corresponding regression lines (dashed lines) with significance levels of their slope coefficients; * denotes statistical significance.

**Figure 10:**
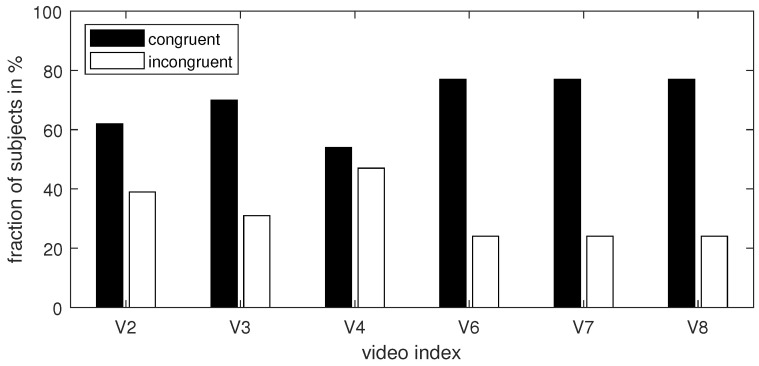
Percentage of subjects with congruent and incongruent trend directions.

To enable time analysis, we constructed a global (i.e., averaged over subjects) regression line for each video event. We tested the significance of the regression line slope using a one-sample *t*-test. The trend lines generated for each video event are depicted in Figure 9. As shown in the figure, a statistically significant trend of consumer engagement was observed for six out of eight videos. The slope of the regression line was negative for four videos (V2, V3, V4, and V8) and positive for two videos (V6 and V7). To determine how many individual results supported these global findings, we constructed individual trend lines for each subject and each event for those six statistically significant global regression lines. Table 6 and Figure 10 summarize this analysis and provide the number of subjects whose trend lines had the same direction as the global regression lines from Figure 9. As observed in the table, not all subjects responded similarly to each video. Generally, a more consistent response was observed for videos with positive trends.

**Figure 11:**
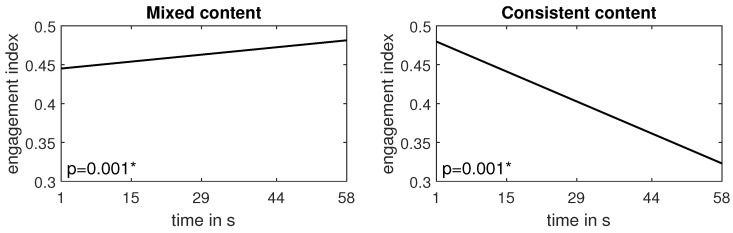
The trend lines averaged over three videos with mixed emotional content (on the **left**) and five videos with consistent emotional content (on the **right**); * denotes statistical significance.

Figure 9 illustrates a much more pronounced distinction between videos with mixed and consistent content compared to the global analysis presented in Figure 7. We can directly observe that for all videos with statistically significant regression line slopes, the trend is negative for consistent content and positive for mixed content. To reinforce this conclusion, we performed an analysis similar to that conducted for global comparison (Figure 7); i.e., we compared the trend averaged over all videos of consistent vs. mixed content. As each video had a different duration, we could not perform this task directly by averaging the trend lines. Instead, we used the coefficients of the regression lines presented in Figure 9 and recalculated the trend lines for the same number of samples for each video. To determine a fair number of samples, we computed the mean length of all videos. Next, we averaged the trend lines for videos with mixed (V1, V6, and V7) and consistent (V2, V3, V4, V5, and V8) contents and obtained the trend lines presented in Figure 11. As shown in the figure, the slope of both trend lines was statistically significant. It is worth noting that the slope of the negative trend found for videos with consistent content was far more prominent than the positive trend found for mixed content. This is quite reasonable since, although it is challenging to sustain consumers’ engagement for an extended period, it is extremely easy to lose their attention at any moment.

## 4. Discussion

Strategies developed to keep the user involved in video content often tend to evoke a specific change in a consumer’s attitude or emotion. Most research in this area has been focused on individual emotions [57], behavioral engagement [12], and consumer attitudes [14]. However, recent studies have shown that this trend may not always be the most effective. For example, research on willingness to watch videos [10] found that content focused on evoking basic emotions did not increase user engagement and did not reduce ad-skipping rates. The results of our study support these findings. Regardless of whether the video had a strongly positive (happiness) or negative (sadness) emotional load, the initial short-term increase in user engagement was followed by a gradual decrease. This effect can be explained by the phenomenon of emotional habituation [18], where the response to continuous stimuli decreases [17]. In our research, the habituation to positive content occurred faster (after 4–5 s for V3 and V8) than to negative (sad) content (after 12–13 s for V2). This result is consistent with earlier studies showing reduced emotional habituation for signals that inform users about negative or dangerous situations [20]. For these types of signals, the habituation phenomenon is reduced but nevertheless also exists [19].

Our study also showed that for videos with mixed emotional loads, the subjects’ engagement fluctuated over time but maintained an upward trend. This trend persisted despite the fact that these videos were much longer than those containing a single emotional message. Similar patterns were reported in earlier studies. This upward engagement trend may be due to the fact that mixed emotional messages are considered less tedious and boring, and therefore do not evoke the intention to skip ads, which is often observed in response to boring content [7].

Content that induces mixed affective experiences has the potential to deliver better performance than pure positive content. This better performance may be a result of higher arousal levels related to higher engagement, as low arousal levels have been reported to lead to lower content performance [6]. Additionally, videos with a mixed emotional message have a greater impact on post-message behaviors than videos containing a single emotion [29].

As previously mentioned, the length of videos with mixed emotional messages used in our experiment did not negatively impact the subjects’ engagement. Our study showed that while all short videos with consistent emotional messages had a declining engagement trend, videos with mixed messages were longer but had a rising engagement trend. Two videos presented a statistically significant trend, while one video presented an increasing but not statistically significant trend. Although video V7 was between three and thirteen times longer than the others, it was able to maintain the participants’ attention for the entire duration of three and a half minutes. These results suggest that using content loaded with mixed emotions can allow for the extension of video length, resulting in higher ad persuasion [11]. This greater persuasion may be a result of the integration of positive and negative emotions [26].

The ability to extend the duration of video content and keep users engaged for longer is a potentially valuable opportunity. However, it also poses a challenge for in-video marketing due to observed consumer annoyance and the intention to skip longer ads [7], perceived as disruptive for goal-oriented searches [9,10]. Negative responses to longer videos have resulted in marketers shortening video content to achieve an acceptable maximal user length [7]. Currently, the duration of marketing video content is typically as low as fifteen or even six seconds [7]. However, this strategy has a severe consequence: short videos have lower potential to elicit emotions or remain in memory. On the other hand, longer videos have better effectiveness and enhance recognition [8]. Advertisements that are shortened to fifteen seconds can deliver similar results to thirty-second spots in terms of awareness and brand recall but have lower potential for persuasion and emotion [11]. Strategies based on mixed emotions can help increase video length, as engaged users are more likely to watch video content and not skip it as quickly as in the case of single-emotion videos [4,5].

## 5. Conclusions

The presented study provides evidence that the type of emotional content significantly influences user engagement in online video ads. Consistent emotional messages tend to lead to habituation, manifested by users’ rapid-onset boredom and decreased involvement in watching presented messages. The results reported in the paper show that, on average, videos with mixed emotional messages tend to achieve higher effectiveness, measured by engagement duration, than those with consistent emotional messages. Moreover, for videos with a consistent emotional load, user involvement decreases with time.

The results of our research suggest that designing marketing content that evokes various emotional states in users leads to maintaining their involvement for extended periods, as opposed to content with a consistent emotional message. On the other hand, the study also suggests that advertising materials with a consistent emotional message should be significantly shorter than those with a mixed emotional message to achieve the same message effectiveness.

The presented study raises questions about the structure of emotional messages, including the sets of emotions used, their sequence, and their duration within videos. Future research should also explore the impact of mixed emotional content on final consumer decisions in a field experiment.

## Figures and Tables

**Table 1:** Subject demographics.

Subject	Age	Gender	Position	Nationality
S1	22	Male	Student	Polish
S2	24	Male	Student	Ukrainian
S3	24	Female	Student	Ukrainian
S4	24	Female	Student	Polish
S5	33	Male	Academic	Polish
S6	57	Male	Academic	Polish
S7	22	Female	Student	Ukrainian
S8	45	Male	Academic	Polish
S9	23	Male	Student	Polish
S10	42	Male	Academic	Polish
S11	23	Female	Student	Polish
S12	38	Female	Academic	Polish
S13	21	Male	Student	Ukrainian

**Table 2:** A short description of the videos watched by the participants.

Id	Description
V1	V1 promotes an internet auction platform. The narrative commences on a rainy day when a man and their young daughter come across a little puppy drenched in water. Subsequently, the video depicts the impact of the dog’s growth on the man’s life, presenting a series of scenes displaying the damage caused by the puppy. The video ends when the father, moved by the close bond between the dog and their daughter, decides to give a home to the dog. The emotional tone of the video changes twice; the opening and ending are sad, while the middle section comprises a blend of surprise and anger.
V2	V2 is a part of a social campaign that sheds light on the deplorable conditions in which African mothers give birth to their children. Although the subject of the video is sad, the message is conveyed mainly through narration, which diminishes its impact. The video is promoted as a part of a charity campaign.
V3	V3 promotes bags from a renowned fashion brand. The black and white video, with subtle red accents, presents a scantily clad woman posing sensually on a chair with a different bag in each scene. While the advertisement is sensual, it lacks excitement.
V4	V4 promotes fruit drinks. The ad shows colorful, flawless fruits and happy people, conveying a clear emotional message—pure joy.
V5	V5 conveys a social message, urging support for a foundation fighting leukemia. The video features a young boy narrating their struggle with the disease for the majority of the video (3/4), while the final part displays information on the disease and support options. Although the video is sad, it is also slightly dull.
V6	V6 is a part of a social campaign. The story takes place on Christmas Eve when a young boy unwraps his gifts. The atmosphere is joyful and loving; the parents chat cheerfully, and everyone is smiling. Among the presents is a cute puppy, which is shot with a gun by the father at the end of the video.
V7	V7 is a part of a social campaign against femicide. It depicts a woman regularly being beaten by her husband and seeking help from various institutions, including the police, church, and family. The emotional tone conveyed by the video is relatively uniform—fear, sadness, and anger—but the intensity of each emotion varies significantly throughout the video.
V8	V8 promotes ice creams. The video comprises several scenes displaying young people happily dancing with ice creams in their hands, creating a positive atmosphere throughout the entire ad.

**Table 3:** The details of the videos used in the experiment. The expected emotions were established via the exclusive analysis of the soundtrack of the underlying video.

Id	Name	Length (s)	Expected Emotions	Type of Emotional Content
V1	What are you looking for in your dreams? [47]	84 (−5)	Sadness, surprise, anger	Mixed
V2	UNICEF Africa, help children in Mali [48]	28	Sadness	Consistent
V3	Unlocked in MYCALVINS [49]	31	Calmness	Consistent
V4	H2Owoc. Without sugar. Without comparison [50]	15	Happiness	Consistent
V5	DKMS Foundation; Leukaemia [51]	78 (−15)	Sadness	Consistent
V6	A dog is not a toy [52]	60 (−5)	Happiness, calmness, fear, anger	Mixed
V7	STOP femicide [53]	231 (−25)	Fear, sadness, anger	Mixed
V8	Keep Summer in Your Heart [54]	34	Happiness	Consistent

**Table 4:** The number of subjects who reported each type of emotion for each video.

	Calm	Happy	Surprised	Excited	Sad	Fear	Angry	Disgust	Total
V1	0	7	7	0	8	0	0	0	22
V2	3	0	0	0	11	0	2	0	16
V3	10	0	2	3	0	0	0	1	16
V4	7	7	0	0	0	0	0	0	14
V5	2	4	2	0	10	0	0	0	18
V6	0	3	10	0	9	3	7	1	33
V7	0	0	3	0	11	7	10	9	40
V8	7	6	0	0	0	0	0	0	13

**Table 5:** The results of the pairwise comparison tests; asterisks denote pairs of video events with significantly different means (*p*-value < 0.01).

	V1	V2	V3	V4	V5	V6	V7	V8
V1		*			*	*	*	*
V2	*							
V3					*		*	
V4								
V5	*		*			*		
V6	*				*		*	
V7	*		*			*		
V8	*							

**Table 6:** Number of subjects whose trend line corresponded/did not correspond to the general tendency; pos—positive, neg—negative tendency.

	V2	V3	V4	V6	V7	V8
	pos	neg	neg	pos	pos	neg
No. of subjects with a congruent trend direction	8	9	7	10	10	10
No. of subjects with an incongruent trend direction	5	4	6	3	3	3

## Data Availability

The data presented in this study are available on request from the corresponding author. The data are not publicly available due to privacy reasons.
